# Mitochondrial replacement techniques: egg donation, genealogy and eugenics

**DOI:** 10.1007/s40592-016-0059-x

**Published:** 2016-07-28

**Authors:** César Palacios-González

**Affiliations:** Centre of Medical Law and Ethics, The Dickson Poon School of Law, King’s College London, Strand, London, WC2R 2LS UK

**Keywords:** Mitochondrial replacement techniques, Mitochondrial donation, Maternal spindle transfer, Pronuclear transfer, Tri-parenthood, Three parent babies, Three parent IVF

## Abstract

Several objections against the morality of researching or employing mitochondrial replacement techniques have been advanced recently. In this paper, I examine three of these objections and show that they are found wanting. First I examine whether mitochondrial replacement techniques, research and clinical practice, should not be carried out because of possible harms to egg donors. Next I assess whether mitochondrial replacement techniques should be banned because they could affect the study of genealogical ancestry. Finally, I examine the claim that mitochondrial replacement techniques are not transferring mitochondrial DNA but nuclear DNA, and that this should be prohibited on ethical grounds.

## Introduction

Mitochondria are organelles which provide the energy necessary for cells to work properly. Two characteristics of mitochondria are that they are only inherited via the maternal line and that they possess their own DNA. Human diploid cells are comprised of nuclear DNA, which accounts for around 99.9 %, and mitochondrial DNA, which accounts for the other 0.1 % (Taylor et al. 2001).

Diseases caused by mitochondria not working properly have been named ‘mitochondrial disease’. Mitochondrial disease “can be caused by either problems in the genes in the nucleus affecting mitochondrial function, or by problems in genes within the mitochondria themselves” (Nuffield Council on Bioethics 2012, p. vii). Mitochondrial *DNA diseases* (henceforth mtDNA diseases) are not one disease but a group of neuromuscular diseases that range in their effects from mild to devastating. They cause heart and major organ failure, dementia, stroke, blindness, deafness, infant encephalopathy, and premature death (Department of Health 2014).

At present, there is no cure for mitochondrial diseases: the only way to treat them is to tackle their symptoms in an effort to increase patients’ overall wellbeing. Because of this, a lot of thought has been focused on how women affected by mitochondrial diseases could have *genetically related* children that do not inherit these diseases. Recent research has devised two different techniques which prevent the passing of diseased mitochondria to further generations, while maintaining a direct genetic link between the intending mother and the child: maternal spindle transfer and pronuclear transfer. These solutions *do not tackle all mitochondrial diseases*: they are effective for a subset in which there are problems within the *genes of mitochondria itself* (Craven et al. 2010; Tachibana et al. 2009; Yabuuchi et al. 2012). From this point onwards, I will only concentrate on mtDNA diseases.^1^ Even when maternal spindle transfer and pronuclear transfer—techniques that have been popularly named ‘*mitochondrial replacement techniques*’ (henceforth MRTs)—could help women to have genetically related children free from the mtDNA diseases, there does not exist total support for them. Françoise Baylis and Stuart A. Newman have raised a number of important ethical objections against MRTs (Baylis 2013; Newman 2013, 2014a, b).

In this paper, I will examine three of the objections that these two authors have advanced and I will show that they are found wanting. I will also prove that even if we accept Baylis’s and Newman’s positions, for the sake of argument, we would have to accept that certain instances of MRTs are morally *unproblematic*. It is important to address the objections that Baylis and Newman advance for three reasons: both are important commentators on the ethics of reproductive techniques, which means that their positions could impact policymaking processes; they have advanced objections against MRTs that have been, for the most part, underexplored; and finally doing so shows that the current UK regulatory stance,^2^ which allows MRTs, can be successfully defended against these specific attacks (HFEA Regulations 2015).

The first argument that I will examine asserts that research surrounding MRTs should not be carried out because of possible harms to egg donors. Next I assess if MRTs should be banned because they could affect the study of genealogical ancestry. Finally, I examine the claim that MRTs do not transfer mitochondrial DNA but nuclear DNA, and that this should be prohibited on ethical grounds.^3^ Before I start examining the arguments against MRTs, I will briefly describe both techniques.

## Mitochondrial replacement techniques

As previously stated, maternal spindle transfer and pronuclear transfer are the two techniques envisaged to aid women afflicted by mtDNA diseases to have genetically related children free from mtDNA disease. In maternal spindle transfer, assisted reproductive techniques are used to obtain oocytes from the prospective mother and a donor. The oocytes from the donor possess healthy mitochondria, while those from the mother contain diseased mitochondria. The chromosomes, which are found on one side of the egg in a spindle-shaped group, from the donor’s oocyte and the mother’s oocyte are then removed. Afterwards, the mother’s chromosomes are transferred to the now enucleated donor’s oocyte. The reconstructed oocyte now has healthy mitochondria and can be fertilised in vitro before being transferred into the mother or a surrogate. If the chromosomal carryover does not cause significant mitochondrial heteroplasmy,^4^ the healthy mitochondria of the reconstructed oocyte will be passed down via the maternal line to subsequent generations, cutting off the mtDNA disease’s transmission. Finally, the donor’s chromosomes and the mother’s enucleated oocyte are discarded (Tachibana et al. 2009; Nuffield Council on Bioethics 2012).

In pronuclear transfer, two zygotes are created in vitro. One of them is created with the prospective parents’ sperm and oocyte (or a sperm from a donor and the mother’s oocyte), and the other one with a donated oocyte and the father’s (or donor’s) sperm. During the first hours, after the sperm has fertilised the oocyte, the nuclear material of both parents is enclosed in different membranes that are called the male and female pronuclei. At day one in the development, and prior to their fusion, the two pronuclei are removed from both zygotes. The pronuclei that were housed in the cell produced with the donor’s oocyte, along with the now enucleated cell that was produced with the mother’s oocyte, are discarded. Afterwards, the intending parents’ (or donor’s and mother’s) pronuclei are transferred to the enucleated cell produced with the donor’s oocyte. The reconstructed zygote is then transferred into the mother or a surrogate (Craven et al. 2010; Nuffield Council on Bioethics 2012). Both techniques allow parent(s) to have genetically related mtDNA disease-free children (supposing that the techniques work and that there is no significant mutant mitochondria carryover during the procedures, which would allow for heteroplasmy to the point of the clinical expression of the disease to occur).

One question that arises at this point is whether it is accurate to call these techniques *mitochondrialreplacement techniques*, if in fact what we are transferring is *nuclear* DNA. Newman maintains that calling them MRTs “has been instrumental in easing the way to public acceptance” but that this name is scientifically inaccurate (Newman 2014a). The terms ‘maternal spindle transfer’ and ‘pronuclear transfer’ are scientifically accurate, so we cannot claim that they are misnomers. However, we have to accept that according to how the techniques should scientifically be described, by means of their common denominator, the *umbrella* term ‘MRTs’ *is* a misnomer, since we are not transferring or replacing mitochondria. Despite this, throughout the text I will use the acronym MRTs when discussing *both* techniques. The justification for doing so is twofold. First, at this stage of the debate the term MRTs has secured a foothold in the ethical discussion of both techniques. Second, and most important, the strength of my counterarguments is not affected by the scientific imprecision of the term. We can replace the term ‘MRTs’ with any other umbrella term and the strength of my counterarguments remains the same. If the reader believes that using the acronym MRTs has a harmful propagandistic effect, then I suggest that every time she reads ‘MRTs’ she mentally replaces it for the less alluring term ‘Nuclear DNA Replacement Techniques Employed to Avoid mtDNA Disease’.

## Harms to egg providers

In her article “The Ethics of Creating Children with Three Genetic Parents”, Françoise Baylis (2013) advances two objections against MRTs based upon potential harms to egg donors, because both techniques require egg donation from women with healthy mitochondria. The first objection asserts that the harm–benefit ratio, when donating oocytes for MRTs, is not favourable enough for pursuing donation. The second objection is that the possible coercion and exploitation of oocyte donors is reason enough for opposing it, thus ruling out mitochondrial replacement.

### Egg donation and the harm–benefit ratio

Baylis, rightly, notes that women who donate oocytes endure daily hormone shots over a span of several days, and that these are uncomfortable, painful, and possess mild to severe medical risks that could lead to

Cramping, abdominal pain, nausea, vomiting, bloating, mood changes and irritability (…) [and more seriously] rapid weight gain and respiratory difficulty, damage to the other organs such as the bladder, bowel and uterus, decreased fertility, infertility and life-threatening haemorrhage, thromboembolism and ovarian, breast or colon cancer. Potential psychological harms include significant stress and sequelae. Baylis (2013, p. 532)

With this clinical panorama as a background, Baylis argues that given that the ‘real’ benefit of enduring such inconveniences (having a child) is not going to be enjoyed by the woman who suffers them (the donor), a harm–benefit calculation would conclude against donating oocytes for MRTs procedures:

The egg provider bears the potential harms of hormonal stimulation and egg retrieval; her only potential benefits are emotional (a good feeling from an act of altruism) and possibly financial (if payment is involved). Baylis (2013, p. 533)

Here Baylis appears to be claiming two things: that the donor *does not benefit enough* from the donation, and that she *suffers more harm* than the egg recipient. There are various problems with this objection. The first is a problem of framing: in her article, Baylis fails to mention that the prevalence of *serious* complications is very low (Delvigne 2009; Maxwell et al. 2008). In fact, severe ovarian hyperstimulation syndrome, according to the World Health Organization, occurs in only up to 0.2–1 % of all cycles in assisted reproduction (Binder et al. 2006). Acknowledging this is important in order not to overstate the medical risks that oocyte donation entails, which is not to say that egg donation is not invasive, uncomfortable or painful.

The second problem is that if we accept her stance on what counts as an acceptable harm–benefit ratio, then we would have to rule out as morally permissible other types of organ donation (Johnson 2013). For example, we would have to accept that cases in which ‘strangers’ decide to make live kidney donations are immoral, and this is absurd (Praagh 2014). We would have to do so because there is no *benefit* to the donor other than an emotional benefit and, sometimes, a compensation for direct receipted expenses. At this point, we can identify the mistake in Baylis’s reasoning: the assumption that there should be some kind of quasi-symmetrical harm–benefit ratio between donor and recipient for donation to be morally permissible. A quasi-symmetrical harm–benefit ratio between donor and recipient is not a *necessary* condition for establishing the morality of pursuing bodily donations that possess statistically low medical risk.

Finally, even if we assumed, for the sake of argument, that the harm–benefit ratio relationship that Baylis endorses is appropriate for normatively assessing donation, she ignores the fact that we could frame egg donation as a kind of supererogatory action. This type of action is good to do, but it is not bad not to do it. What I mean here is that even when there is no duty whatsoever to donate eggs doing so would be morally commendable given that this would enable other people to carry on with their reproductive goals. Egg donation for the purposes of MRTs can be seen as an action that goes “beyond the call of duty” (Urmson 1958).

### Exploitation and oocyte donation

The second objection that Baylis advances is that women, especially those who are economically disadvantaged, are at risk of being coerced and exploited for their eggs [as has been reported in developing countries such as India (Durgesh 2014)], and this is reason enough to oppose MRTs, in as much as they need donated oocytes.^5^

It goes without saying that I, and every reasonable person, completely agree with Baylis in that there should be strict legal safeguards against the coercion and exploitation of women. Now, it should be noted that under Baylis’s account oocyte donation seems not to be inherently exploitative; this means that oocyte donation is not in itself morally objectionable but rather that it is objectionable *only when* women are coerced or exploited. Therefore, as long as no exploitation or coercion occurs this argument cannot be invoked as an objection to oocyte donation for MRTs, or other assisted reproductive techniques. Furthermore, Baylis’s argument loses its strength when legal safeguards against the exploitation of women are appropriate, effective and in place. The regulation of MRTs could help fight against the coercion and exploitation of women who donate oocytes if regulations included adequate provisions regarding the circumstances in which oocyte donation is permissible, and the conditions under which it should be carried out. For example, procedures should be established to eliminate undue medical influence upon women who are considering oocyte donation, and to determine what type of compensation, if any, women should receive for oocyte retrieval.^6^^,^^7^

There are cases of oocyte donation that are morally unproblematic from a coercion/exploitation point of view, and we do not regard as morally necessary that the harm–benefit ratio of this type of bodily donation be quasi-symmetrical for donors and recipients. This being the case, we can affirm that Baylis’s first two objections fail to provide sufficient reasons for banning oocyte donation, and thus impede research into MRTs or their application.

## Genealogical ancestry

Baylis has also argued that “[m]itochondrial replacement technology represents a potential threat to genealogical research using mtDNA analysis, as it would obscure the lines of individual descent, thereby providing a false or confusing picture” (Baylis 2013, p. 533). It is true that MRTs could affect the interests of those who place value, either for identity purposes or academic research, on genealogical information that can be traced by mtDNA, but we need to further investigate if this is reason enough not to pursue such procedures.

First, it should be noted that it is open to further *empirical investigation* what degree of mitochondrial heteroplasmy, caused by MRTs, would be necessary to track down both genealogical lines (donor’s and mother’s). If very mild mitochondrial heteroplasmy is sufficient to identify both genealogical lines then the use of MRTs for reproductive purposes, as it caused such heteroplasmy, would *not* necessarily imply the obscurement of genealogical lines. If this were the case then Baylis’s objection would only apply to those cases of MRTs that *did not* cause mitochondrial heteroplasmy, or that caused it to the extent that the genealogical line of the woman who desires to be a genetic mother could not be traced.

Second, even if we accept, for the sake of argument, that genealogical research is valuable enough to legitimately constrain people’s reproductive rights, this does not entail that MRTs should be ruled out as reproductive options: we could counter the problem by requiring both sex selection^8^ and disclosure in order to avoid ‘obscuring’ genealogical information.

We could require women, or couples, to select only for males when MRT conceiving (Bredenoord et al. 2010; Appleby 2015),^9^ as doing so would assure that genealogical research reliant upon mtDNA is only ‘disrupted’ in the first generation of MRT-conceived men. All future mtDNA genealogical research on the family lines of MRT-conceived men would not be affected, as mitochondria are inherited via the *maternal* line.

However, while this answers the genealogical concern about future generations, a problem remains: those men born from MRT conceptions may have an ‘obscured’ perception of their genealogical history. In order to address this issue, we could additionally* require* that parents disclose to their children that they were MRT conceived and that this was stated on their medical records (or birth certificates). With this requirement, all MRT-conceived men with an interest in their genealogical information would have an accurate picture of their genetic origins; and scientists would be in a better position for doing research.

John Appleby has put forward three additional reasons, *not based upon arguments around genealogical research*, for favouring disclosure in the context of MRT conception: (i) children conceived this way need not be subject to the stress and anxiety of worrying about having the same mtDNA disease as their mothers; (ii) the MRT-conceived person’s own medical welfare; and (iii) the ability to report problems to healthcare professionals for the sake of future generations (Appleby 2015, p. 507). Although these reasons are not directly related to genealogical research, they further weaken Baylis’s objection (when considered in addition to only male selection), by building a case for revealing their status to those that were MRT conceived.

If we consider disclosure and sex selection together, then an argument based on the ‘obscurement’ of genealogical information loses its grip: firstly because MRT-conceived men would have a clear picture of their mtDNA status, and thus of how their conception affects the genealogical information obtainable through their mtDNA. Furthermore, they can access genealogical information by means of their mother’s mtDNA, if she granted her consent for doing so. Secondly, any ‘disruption’ caused by MRTs would be ‘contained’ by the first generation of MRT-conceived men, as those who follow would inherit their mitochondria via the maternal line.^10^

A third attack on Baylis’s objection shows that the use of MRTs falls within our reproductive rights, and that these rights trump any kind of genealogical research claim that scientists may have. If we accept that the use of assisted reproductive techniques, for example IVF, falls under the scope of our reproductive rights (even if such rights only entail the *non*-*interference* of third parties in our reproductive choices), then we have to accept that MRTs also fall within our reproductive rights. We have to accept this because MRTs, just as other assisted reproductive techniques, both allow us to fulfil our reproductive aims and do not harm the children produced by them.^11^ Now, the goals of *scientific research* trump over people’s reproductive rights only under the most extreme circumstances. At this point we need to note two things. First, the aims of genealogical research are ‘obstructed’ by MRTs in only a handful of cases. It is expected, by the UK Department of Health (2014, p. 38), that only 10 children would be the product of MRTs every year. Second, and more importantly, the *benefits* of genealogical research that would be forfeited because some people employ MRTs are so *insignificant* that they do not satisfy the *extremeness* condition for curtailing people’s reproductive rights. Finally, Baylis herself concedes that her objection is not very strong, admitting that while “preserving the ability of DNA genealogists to do their research may not be a high social priority, it is nonetheless deserving of consideration recognizing that little is known at this time about personal and cultural attitudes towards mtDNA” (Baylis 2013, p. 533).

I have shown that genealogists do not have a strong enough claim to trump people’s reproductive rights, but what about parental attitudes towards genealogical information? Although *empirical* data are missing to support the following claim I contend that it is commonsensical to assume that if faced with a choice between using MRTs or conceiving a child at risk of developing a mtDNA disease most people would choose MRTs despite the concern of obscuring genealogical information. It seems quite implausible that people (present and future) would forgo having mtDNA disease free children, in order that their children with mtDNA diseases are be able to know, through mtDNA, their ‘un-obscured’ ancestry. It is important to remember that people could access their mtDNA genealogical information matrilinealilly, if needed, and that people with mtDNA diseases contemplate MRTs because mtDNA diseases cause pain, are debilitating, and life shortening.

## Are we transferring mitochondria or nuclear DNA?

Stuart A. Newman, Professor of Cell Biology and Anatomy at New York Medical College, has claimed that those advocating MRTs are intentionally misguiding the public debate by using the name ‘mitochondrial transfer’ or ‘mitochondrial replacement’. According to him, scientists and media commentators are portraying the techniques as only transferring *mitochondrial DNA*, when in reality what is being transferred is *nuclear DNA*. By presenting MRTs as if they were only transferring 0.1 % of the total DNA material (mtDNA is composed of only 37 genes), they are trying to win wide public support while skewing the debate:

It is clear, however, that much more than mitochondria is being transferred or donated in MST [maternal spindle transfer]. This is obscured in most reports on the subject, even in scientific journals. A recent report in the journal *Nature* states, ‘The technique [combines] genetic material from a mitochondria donor, the mother who provides the nucleus and a father.’ To use the emotive term ‘mother’ only for the donor of the maternal set of chromosomes downplays the unique biological role of the oocyte and of the woman who contributes it. It has the further effect of endorsing the false assertion of MST’s advocates that the procedure comes down to the transfer of a few (i.e. the mitochondrial) genes. What is actually being transferred are 20,000 or so genes provided by the chromosome donor. (Newman 2014b)

While it is true that the term ‘mitochondrial replacement techniques’ is a misnomer [as discussed in Sect. (2)], the important question is—does the amount of DNA being transferred matter? Newman believes it does. He maintains that large-scale genetic material transfer for reproductive purposes (i.e. MRTs) should not be attempted because this would open “the door to routine applications of germline (i.e. inheritable) gene modification.”Newman (2014b).

Newman’s objection against MRTs is that once we allow, for reproductive purposes, the transfer of nuclear DNA, with its “20,000 or so genes”, we would also be committed to allowing other germline modifications. He pictures a world where once we have accepted MRTs, we would also accept a kind of ‘selectionist’ eugenics:

The procedures described [MRTs], currently under evaluation by the British Human Fertilisation and Embryo Authority (HFEA) for the prevention of “mitochondrial diseases,” would carry profoundly negative implications for the future of the human species were they ever implemented, and thus warrant much wider concern than what they have attracted up to now. In particular, they will facilitate a new form of eugenics, the improvement of humans by deliberately choosing their inherited traits. (Newman 2013)

There are three possible ways in which to address Newman’s objection. The first is to examine whether the conclusion (i.e. becoming a ‘selectionist’ eugenics society) is something that we would morally want to avoid. While historical forms of eugenics (i.e. coercive state mandated positive and negative eugenics aimed at improving the human ‘stock’) are morally appalling, the case for *liberal eugenics* has been successfully defended by Nicholas Agar (2004). According to Agar, liberal societies ought to respect people’s conception of human excellence (i.e. liberal societies should allow for a pluralistic view of what counts as a good life), and this respect should extend to the way people decide to make their children. He maintains that there should be *two restrictions* to liberal eugenics: (i) biotechnologies should not be used to solve complex social problems (e.g. racism) and (ii) children born from such biotechnologies ought not to be harmed. If, as I contend, Agar has shown that liberal eugenics is morally defensible then Newman’s objection is unsuccessful if MRTs are not aimed at solving a complex social problem, which they are not, and if MRTs do not harm the children produced by them.

An important question to answer here is whether MRTs can cause harm to the children conceived this way? MRTs *cannot* harm these children, as for an action to harm someone it needs to make that person worse off than she would have been had the action not taken place (Feinberg 1986). MRTs cannot make anyone *worse off*, because without them being employed these individuals would not have existed in the first place: this is known as the non-identity effect (Boonin 2008; Lawlor 2015). MRTs *determine* the *identity* (identity in the sense of what genetic material individuates a biological being) of who would be ‘brought’ into existence by affecting which gametes will fuse. For example, the use of MRTs affects the *timing* of conception, and thus they affect which particular sperm will fertilise the intending mother’s oocyte. Ultimately, the choice is not between a life where one was affected by MRTs and another where one was not affected by MRTs. The decision is between existence and non-existence.

The only exception to this is when we have preselected a sperm and egg, for our reproductive purposes, before we decide to use MRTs. In those scenarios, the use of MRTs *does not affect* which sperm and oocyte will fuse, and so it is true that things *could have been otherwise* for these MRT-conceived children.^12^

The second way in which we can address Newman’s objection is to show that *in practice* (i.e. in the real world) the acceptance of MRTs does not send us down the slippery slope of accepting all germline modifications. This can easily be done by pointing out how the UK has regulated on MRTs [and legislates in relation to assisted reproductive techniques (HFE Act 1990, as amended 2008)], and how the US Institute of Medicine has reacted to the possibility of MRTs. The UK has created legislation on assisted reproductive techniques on a case by case basis. Therefore, against this context, the claim that to accept MRTs is to mount the slippery slope is misleading (Lords Discusses Mitochondrial Donation—News from Parliament 2015).

The US Institute of Medicine, on the other hand, has established a special committee to discuss the ethical and policy questions around MRTs (Institute of Medicine of the National Academies 2015). This indicates that the consideration or acceptance of MRTs does not entail, *in practice*, that other germline modifying techniques, or DNA transferring techniques, would be *automatically* and *unreflectively* accepted. For example, while MRTs have been accepted in the UK, somatic cell nuclear transfer (i.e. cloning) for reproductive purposes has not been so. Regardless of whether the UK’s decision regarding reproductive cloning is cogent, this shows that *in practice* the fact that one technique is accepted does not entail that all other ‘similar’ techniques would be accepted.

We can interpret Newman’s argument, alternatively, as one regarding (theoretical) moral consistency, and not one aimed at predicting how germline modifications would be regulated in the real world. According to this interpretation, if we conclude that MRTs are morally permissible then consistency requires that we regard *all other* germline modifications as morally permissible. Even if we favour this reading of Newman’s argument, it will not get us far: there is a distinction to be made between the in general moral acceptability of germline modifications, and the moral acceptability of *particular* germline modifications. It does not follow from the fact that in general we accept germline modifications as morally permissible that *all instances* of germline modifications are morally permissible. There are particular instances of germline modification that would undoubtedly be immoral to carry out. For example, inserting a gene into an early embryo whose only function would be to cause unbearable pain, if this was ever possible. This is a germline modification because all future generations will inherit the pain-causing gene, and it is clearly immoral to carry out such an intervention. All germline modifications need to be ethically assessed on a case-by-case basis.

Finally, a third way to address Newman’s objection is to point out that even when MRTs are a kind of large-scale genetic material transfer, they *do not necessarily amount to germline modifications*. Neither maternal spindle transfer nor pronuclear transfer *necessarily* amounts to germline modifications if we hold that a *necessary condition* for a genetic modification to be regarded as a germline one is that such modification *can* be inherited. Through sperm sorting or preimplantation genetic diagnosis after MRT, we could only select males. This would prevent any possibility that the ‘transferred’ mitochondria could be inherited^13^: the nature of mitochondrial inheritance is such that we can only refer to MRTs as ‘germline-modifying techniques’ when they affect females, by either bringing them into existence or by treating their mtDNA disease, since it is only in this case when the ‘modification’ could be inherited.^14^ Realising this shows that Newman’s germline modification objection is not really that strong.

## Conclusion

In this paper, I have shown that three of the recent objections against MRTs, advanced by Baylis and Newman, are flawed. I have also shown that even if for the sake of argument we accept those objections that are not patently indefensible, they do not rule out *all* instances of MRTs. This last finding is of paramount importance for those operating both under an ethical and legal bio-conservative framework, since it helps them to build a bio-conservative case for MRTs. The fact that these three ethical objections fail is significant from a policymaking perspective. Indeed, this shows that the recent decision by the UK parliament (HFE Regulations 2015) to allow MRTs can be ethically defended against these specific attacks, and therefore helps to pave down the ethical road for other legislatures where MRTs are being discussed, such as the US. 

## Footnotes

1 Another technique (which I will not address here) that has been discussed in relation to tackling mtDNA diseases is cytoplasmic transfer. This technique involves injecting cytoplasm from a donor’s oocyte, with healthy mitochondria, into an oocyte with diseased mitochondria (Brenner et al. 2000).

2 For a discussion on the regulation of MRTs in the UK, see Appleby (2015).

3 It must be clear that in this paper I will not examine *all* the objections that have been presented against MRTs, as I have previously said. For recent works on the ethics of MRTs, which deal with issues of identity, see: Nuffield Council on Bioethics (2012), Institute of Medicine of the National Academies (2016), Wrigley et al. (2015) and Bredenoord et al. (2011). On issues of transgenerational health risks, see: Nuffield Council on Bioethics (2012), Institute of Medicine of the National Academies (2016), Appleby (2015), Baylis (2013), Johnson (2013) and Bredenoord et al. (2010). On issues of disclosure of MRT conception, see: Nuffield Council on Bioethics (2012) and Appleby (2015). On issues of MRTs funding, see: Harris (2015), Harris (2016), Rulli (2016), de Melo-Martin (2017), Harris (2017) and Palacios-González (2017). On issues of first in-human use, see: Nuffield Council on Bioethics (2012), Institute of Medicine of the National Academies (2016) and Bredenoord and Braude (2010). On the possible use of nonhuman oocytes for PNT, see Baylis (2009), and on the anonymity status of the ‘mitochondrial donor’, see Appleby (2016).

4 Deleterious mutations in the mitochondria can be present either in all mitochondria, referred to as ‘homoplasmy’, or only in some mitochondria, known as ‘heteroplasmy’. For recent research on MRTs and mitochondrial carryover, see Hyslop et al. (2016) and Yamada et al. (2016)

5 In future, it might be possible to avoid oocyte retrieval altogether, if scientists are able to derive functional oocytes from human induced pluripotent stem cells (Hayashi et al. 2012; Eguizabal et al. 2011; Palacios-González et al. 2014).

6 In this regard, the UK is a good example. The UK’s HFEA, through its Code of Practice, has established procedures for limiting the possibility that women are exploited throughout the donation process, or coerced into oocyte donation (HFEA Code of Practice 2015, Chpt. 11).

7 While this paper was under review, the US Institute of Medicine of the National Academies (IOM) published its report ‘Mitochondrial Replacement Techniques: Ethical, Social, and Policy Considerations’. In it they recommend that MRTs researchers consider not only current guidance but also *emerging best practices* in determining appropriate compensation for gamete providers, thus effectively improving the gamete donation context: “It would be important for MRT researchers and institutions, in consultation with local review committees or a central IRB, to consider current guidance and emerging best practices in determining appropriate compensation for gamete providers, taking into account the demands placed on a gamete provider by an MRT research protocol” (Institute of Medicine of the National Academies 2016, Sect. 4, p. 17).

8 As previously said, Bredenoord and Braude (2010), Appleby (2015), and The Institute of Medicine of the National Academies (2016) have examined the morality of sex selection for avoiding possible transgenerational risks due to MRTs.

9 It is open to further investigation whether using sex selection for the purpose of allowing genealogical research is open to the same criticism as when used for nonmedical purposes.

10 The IOM report, following Baylis (2013), also found that “[t]he novel combination of mtDNA and nDNA that would result from MRT blurs traditional notions of relatedness in ways that may undermine intergenerational connections and lineage as measured by mtDNA” (Institute of Medicine of the National Academies 2016, Sect. 3, p. 18). It is interesting to note that even when the IOM report’s final recommendation is to choose for males, when carrying out initial clinical investigations on MRTs, they missed that this would *contain* any disruption to the ‘natural’ mtDNA intergenerational connection.

11 There is only one instance of MRTs that could harm the child produced through them (see next section), but this possibility is highly unlikely to ensue.

12 Wrigley et al. (2015) have defended that there is a moral difference between pronuclear transfer and maternal spindle transfer, in that the former is a form of therapy, while the latter is an instance of selective reproduction. They advance that the identity of the person who will be ‘brought’ into existence, in the case where pronuclear transfer will be carried out, is already determined via its genetic endowment and thus we should regard such a technique as therapeutic and morally preferable to maternal spindle transfer. These authors’ assertion, in fact, *is only true* for those cases where we have selected, beforehand, a sperm and egg for our reproductive purposes. This is so because, in all other instances, the identity of who will be ‘brought’ into existence is dependent on the MRTs taking place.

13 It should be noted that this could change if, by means of in vitro gametogenesis, we were able to derive functional gametes of both sexes from *male* iPSC, as previously noted (Palacios-González et al. 2014; Hübner et al. 2003; Kerkis et al. 2007; Lacham-Kaplan et al. 2006). It could happen that a MRT-conceived man decided to use his cells to produce an oocyte. If this occurred, then *all instances* of MRTs could amount to germline modifications. One way of dealing with this issue would be to always require that the other person involved in the reproductive process produced the oocyte (supposing that one of them was not MRT conceived). For example, suppose that a MRT-conceived man was in a same-sex relationship and that they decided to use *in vitro* gametogenesis for their reproductive purposes. If this were the case, then we could require that the non-MRT-conceived man produced the oocyte.

14 The IOM report reached the same conclusion regarding the ‘germline’ status of MRTs: “MRT results in genetic modification of germ cells. Because mtDNA is solely maternally inherited, MRT producing female offspring would constitute heritable genetic modification (germline modification). Although MRT results in genetic modification of germ cells, those modifications are not heritable in males. Thus MRT producing male offspring would not constitute heritable genetic modification (germline modification)” (Institute of Medicine of the National Academies 2016, Sect. 3, p. 8).
